# Genetic diversity and pathogenicity of novel recombinant strains of PRRSV lineages 1.5, lineages 1.8 and lineages 8 in South China

**DOI:** 10.3389/fcimb.2026.1798945

**Published:** 2026-06-30

**Authors:** Xiaopeng Gao, Guangrun Qin, Yuanyuan Fu, Zechang Fan, Limiao Lin, Haishen Zhao, Bohua Ren, Qunhui Li, Yu Wu

**Affiliations:** 1 Key Laboratory of Zoonosis Prevention and Control of Guangdong Province, College of Veterinary Medicine, South China Agricultural University, Guangzhou, China; 2 Wen’s Food Group, Yunfu, China

**Keywords:** epidemiological investigation, pathogenicity, PRRSV, PRRSV lineages 1.5 1.8 and 8, recombination

## Abstract

Porcine reproductive and respiratory syndrome virus (PRRSV), particularly genotype 2 (PRRSV-2), inflicts substantial economic losses on the global swine industry. In China, Lineage 1 of PRRSV-2 has been the dominant epidemic strain since 2016 and is characterized by frequent recombination. This study investigated the molecular epidemiology of PRRSV in Southern China from 2022 to 2024. Among 1, 139 clinical samples from Guangdong, Guangxi, and Hainan provinces, the overall PRRSV positivity rate was 40.6% (463/1, 139). NADC30-like (Lineage 1.8, 49.9%) and NADC34-like (Lineage 1.5, 31.1%) strains were identified as the most prevalent. A novel recombinant strain, designated DJW, was successfully isolated using porcine alveolar macrophages. Whole-genome and GP5 phylogenetic analyses revealed DJW as a triple-recombinant mosaic virus. It possesses a NADC30-like backbone but has undergone recombination with HP PRRSV-like strains in the NSP3–NSP9 region and with NADC34-like strains in the ORF2-ORF6 region. Pathogenicity assessment in piglets demonstrated that DJW infection causes severe clinical signs, including persistent fever, significant viremia, robust viral shedding, extensive lung consolidation, and one death observed in the challenge group (1/5). Our findings highlight the ongoing evolution of PRRSV through recombination.

## 1 Introduction

Porcine reproductive and respiratory syndrome virus (PRRSV) is a highly contagious virus in the family *Arteriviridae* that primarily targets porcine alveolar macrophages (Albina et al., 1998; Music and Gagnon, 2010), leading to multisystemic dysfunction and immunosuppression (Benfield et al., 1992; Ivashkiv and Donlin, 2014). In 2021, the International Committee on Taxonomy of Viruses officially reclassified PRRSV into two distinct species: *Betaarterivirus suid 1* (PRRSV-1, European genotype) and *Betaarterivirus suid 2* (PRRSV-2, North American genotype) (Shi et al., 2010; Kappes and Faaberg, 2015; Brinton et al., 2021). Typical clinical manifestations include reproductive failure in pregnant sows (e.g., abortion, stillbirths, mummified fetuses) (Pejsak et al., 1997; Solano et al., 1997; Neumann et al., 2005), respiratory distress in piglets (Solano et al., 1997; Xu et al., 2010), and cyanosis of the ears (hence the common name “Blue Ear Disease”) (Neumann et al., 2005; Wang et al., 2017). Pre-weaning mortality rates of PRRSV infections can reach up to 70% (Pejsak et al., 1997; Tian et al., 2007).

PRRSV has spread globally since its emergence in the United States in 1987 (Benfield et al., 1992; Collins et al., 1992). The European prototype strain, Lelystad virus, was first isolated in the Netherlands in 1991 (Yoon et al., 1996). Subsequently, the North American prototype strain, VR-2332, was isolated by Collins et al. in the United States in 1992 (Nelsen et al., 1999). These two genotypes share approximately only 60% genomic nucleotide identity, indicating significant genetic divergence (Meulenberg et al., 1993). In China, the first classical PRRSV-2 strain, CH-1a, was isolated in 1996 (Tian et al., 2007). A major outbreak of highly pathogenic PRRSV (HP-PRRSV) infection occurred in 2006 (Zhou et al., 2008), characterized by high fever, high morbidity, and high mortality, resulting in severe losses to the swine industry. Recent PRRSV epidemics in China have shown increasing complexity (Zhang et al., 2016; Gao et al., 2017; Liu et al., 2021). Notably, NADC30-like strains (Lineage 1.8), which have disseminated widely since 2013, have undergone multiple recombination events with HP-PRRSV and other strains (Zhong et al., 2012; Zhao et al., 2015; Zhang et al., 2016). Furthermore, NADC34-like strains, first identified in 2017 (Zhang H. et al., 2018; Zhao et al., 2022), exhibit diverse recombination patterns and have the potential to become new dominant strains.

For over three decades since it was first isolated, PRRSV has caused substantial losses and continue to pose a persistent threat to the swine industry in China. PRRSV is inherently characterized by a rapid mutation rate and high recombination frequency (Wang et al., 2013; Kappes and Faaberg, 2015; Wang et al., 2021). The genetic diversity of PRRSV in China is exceptionally complex, compounded by the continual incursion of imported strains, thereby significantly impeding effective control measures (Papatsiros et al., 2006; Mateu and Diaz, 2008; Renukaradhya et al., 2015). This study conducted a systematic epidemiological investigation to elucidate the prevalent characteristics, predominant strain types, and genetic evolutionary patterns of PRRSV in southern China. The findings would provide a scientific basis for the development of targeted regional prevention and control strategies (Zhou et al., 2021).

## 2 Materials and methods

### 2.1 Processing of clinical samples

#### 2.1.1 Blood samples

Aliquots of whole blood samples were placed into 1.5-mL standard centrifuge tubes and centrifuged at 10, 000 × *g* for 2 min at 4 °C. The serum was separated by aspiration and transferred into sterile EP tubes for subsequent use. All clinical samples (Blood, Tissue and Swab)in this study were collected from the pig farms of Wen’s Group.

#### 2.1.2 Tissue samples

Appropriate amounts of representative tissue samples were minced and placed into sterilized 2-mL EP tubes, each containing two sterile steel beads. The tissues were homogenized using a frozen tissue homogenizer (impact frequency: 50 Hz, 3 cycles). The homogenates were centrifuged at 8, 000 × *g* for 5 min, and the supernatants were collected for further analysis.

#### 2.1.3 Swab samples

Swabs were placed into 1.5-mL EP tubes containing 800 μL physiological saline and vigorously vortexed on a horizontal shaker at 1200 rpm for 5 min. The samples were then centrifuged at 8, 000 × *g* for 2 min, and the supernatants were collected.

### 2.2 RNA extraction

RNA extraction was performed using a Bioer GenePure Pro automated nucleic acid extraction and purification system. Samples were lysed and then transferred to the extraction plate provided with the instrument. The plate was placed into the GenePure Pro system, which automatically completed the nucleic acid extraction and purification processes. The program parameters (including lysis temperature, elution temperature, magnetic bead separation time, and intensity) were set according to the manufacturer’s instructions. The extracted RNA was stored at −80 °C for subsequent experiments.

### 2.3 Reverse transcription polymerase chain reaction, sequencing, and phylogenetic analysis

*ORF5* was amplified using a One Step Reverse Transcription Polymerase Chain Reaction (RT-PCR) Kit (Dye Plus) with primers designed from reference strains (NADC30, NADC34, JXA1, VR2332, and QYYZ). Amplification products were visualized on 1% agarose gels and sent to a third-party laboratory for Sanger sequencing (Sangon Biotech, Shanghai, China).

All PRRSV-positive samples were initially genotyped using a multiplex real-time RT-PCR assay targeting lineage-specific signatures in the ORF5 region. This assay allowed assignment of positive samples to five lineages: NADC30-like (Lineage 1.8), NADC34-like (Lineage 1.5), VR2332-like (Lineage 5.1), HP-PRRSV-like (Lineage 8.7), and QYYZ-like (Lineage 3). For phylogenetic confirmation and detailed genetic analysis, 124 representative samples with lower cycle threshold (Ct) values (<30) were selected for ORF5 Sanger sequencing.

Viral genome sequences were assembled using DNASTAR SeqMan. Phylogenetic trees were constructed on MEGA11 using the maximum likelihood method based on full-genome and *ORF5* sequences. Reference sequences were downloaded from GenBank.

### 2.4 Recombination analysis

Recombination events were analyzed using RDP4. A significant recombination event was defined as that detected by at least six of the seven built-in methods (RDP, Chimaera, BootScan, 3Seq, GENECONV, MaxChi, and SiScan) with a P-value < 0.05. Recombination breakpoints were verified using SimPlot 3.5.1.

### 2.5 Virus isolation and propagation

PRRSV-positive tissue homogenates were filtered (0.22-μm membrane), inoculated into porcine alveolar macrophages (PAMs), and cultured at 37 °C with 5% CO_2_. The cultures were monitored daily until they reached 80% cytopathic effect (CPE), harvested, and then stored at −80 °C.

PRRSV was subsequently propagated in Marc-145 cells cultured in Dulbecco’s Modified Eagle Medium containing 10% fetal bovine serum and 1% penicillin–streptomycin. When CPE > 80%, the culture was freeze–thawed and centrifuged, and the supernatant was collected as virus stock (passage P2). The process was repeated for subsequent passages.

### 2.6 Virus titration

Virus titers were determined on Marc-145 cells placed in 96-well plates. Serial 10-fold virus dilutions were inoculated into quadruplicate wells. After 2 h adsorption, maintenance medium was added. Plates were monitored for CPE for 5–7 days. The 50% tissue culture infectious dose (TCID_50_) was calculated using the Reed–Muench method.

### 2.7 Indirect immunofluorescence assay

Marc-145 cells grown in 6-well plates were inoculated with the DJW strain. When CPE was observed, cells were fixed with 4% paraformaldehyde, permeabilized with 0.3% Triton X-100, and blocked with 2% bovine serum albumin. Cells were incubated, first with the primary antibody at 4 °C for 8–12 h, then with the fluorophore-conjugated secondary antibody at 37 °C for 1 h). Nuclei were stained with 4′, 6-diamidino-2-phenylindole (DAPI). Images were captured through a fluorescence microscope.

### 2.8 Virus purification and transmission electron microscopy

The virus culture supernatant was clarified by low-speed centrifugation (5, 000 × *g*, 10 min) and filtered through a 0.22-μm membrane. The filtered supernatant was ultracentrifuged at 40, 000 × *g* at 4 °C for 3 h. The pellet was resuspended in phosphate-buffered saline and sent to Wuhan Servicebio Technology Co., Ltd. for analysis by transmission electron microscopy (TEM).

### 2.9 Pathogenicity assessment of DJW strain in piglets: experimental design

**Figure 1:**
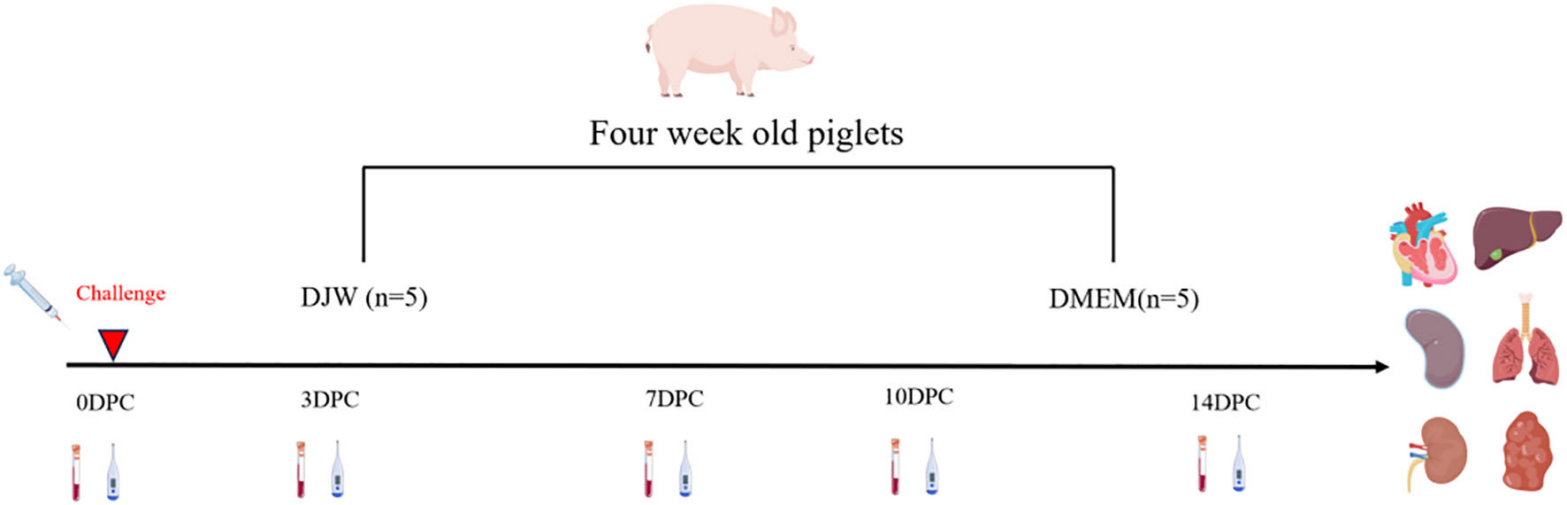
Experimental design for evaluating the pathogenicity of DJW strain in piglets.

Ten healthy piglets, confirmed negative for both PRRSV antigen and antibodies, were selected and randomly allocated into two groups: the challenge group (DJW group) and the control group. Piglets in the challenge group (n=5) were inoculated with a viral suspension containing 2×10^5^ TCID_50_ of PRRSV via intramuscular injection of 2 mL and intranasal instillation of 2 mL, resulting in a total inoculation volume of 4 mL per animal. The control group (n=5) received an equivalent volume of DMEM medium following the same inoculation procedure. All animals were handled identically throughout the experiment to ensure that all conditions, except for the inoculum, remained consistent between groups (**Figure 1**). All piglets used in this study were provided by the farms of the Wen’s Group.

Rectal temperature and clinical signs were monitored daily. Body weight was recorded on days 7 and 14 post-inoculation (dpi). Whole blood, oropharyngeal swabs, and anal swabs were aseptically collected at 0, 3, 7, 10, and 14 dpi for viral load quantification (qRT-PCR) and antibody titer determination to evaluate systemic infection status. Survival rates were recorded throughout the study. On day 14 post-challenge, all piglets were euthanized, and tissue samples—including heart, liver, spleen, lung, kidney, bilateral submandibular lymph nodes, and inguinal lymph nodes—were systematically collected. Viral nucleic acid load in these tissues was measured by quantitative real-time PCR. Additionally, lung tissues were subjected to paraffin embedding, sectioning, and hematoxylin-eosin (HE) staining for histopathological evaluation.

### 2.10 Statistical analysis and data visualization

Statistical significance between experimental groups was assessed using one-way analysis of variance (ANOVA). A two-tailed p-value of less than 0.05 (P < 0.05) was considered statistically significant. All point graphs were generated using GraphPad Prism (v8.0.0). The detected recombination events were further confirmed by SimPlot 3.5.1, which was performed within a 200 bp window, sliding along the genome alignments with a 20 bp step size.

## 3 Results

### 3.1 PRRSV positivity in clinical samples collected in 2022–2024

In total, 1, 139 clinical samples were collected from suspected infected pig farms in 12 cities across three provinces (Guangdong, Guangxi, and Hainan) in southern China between 2022 and 2024. Quantitative real-time PCR detected 463 PRRSV-positive samples, yielding a positivity rate of 40.6%. Of the positive samples, 231 (49.9%) were identified as NADC30-like strains, 144 (31.1%) as NADC34-like strains, 46 (9.9%) as VR2332-like strains, 29 (6.3%) as HP-PRRSV-like strains, and 13 (2.8%) as QYYZ-like strains.

Based on the multiplex real-time RT-PCR genotyping assay, the remaining 339 positive samples (not subjected to sequencing) were assigned to the lineages shown in **Table 1**. To further investigate the genetic evolutionary characteristics of the prevalent PRRSV strains in southern China, *ORF5* in PRRSV-positive samples was sequenced. The amplified products exhibited bands consistent with the expected sizes of the target gene fragments. Phylogenetic analysis of 124 *ORF5* sequences revealed that 74 sequences (59.68%) were clustered within Lineage 1.8, 45 (36.29%) within Lineage 1.5, and 2 (1.61%) and 3 (2.42%) sequences within Lineages 5.1 and 8.7, respectively. No sequences were clustered within Lineage 3 or 8.9.

**Table 1:** Epidemiological survey of PRRSV in South China from 2022 to 2024.

Area		Total count	Positive count	NADC30 -like	NADC34-like	VR2332-like	HP -like	QYYZ-like
Guangdong	Yunfu	124	49	21	19	5	2	2
	Yangjiang	61	20	10	5	1	3	1
	Heyuan	68	18	9	5	2	1	1
	Jiangmen	49	13	5	2	2	3	1
	Shaoguan	53	36	12	19	1	2	2
	Qingyuan	112	41	20	12	7	2	0
	Zhaoqing	114	64	31	19	6	6	2
Guangxi	Hezhou	133	53	27	19	4	2	1
	Yulin	128	44	26	8	5	3	2
	Guilin	65	34	21	9	3	1	0
Hai nan	Qionghai	89	36	24	7	4	1	0
	Danzhou	143	55	25	20	6	3	1
Total		1139	463	231	144	46	29	13

### 3.2 Isolation and culture of PRRSV strains

**Figure 2:**
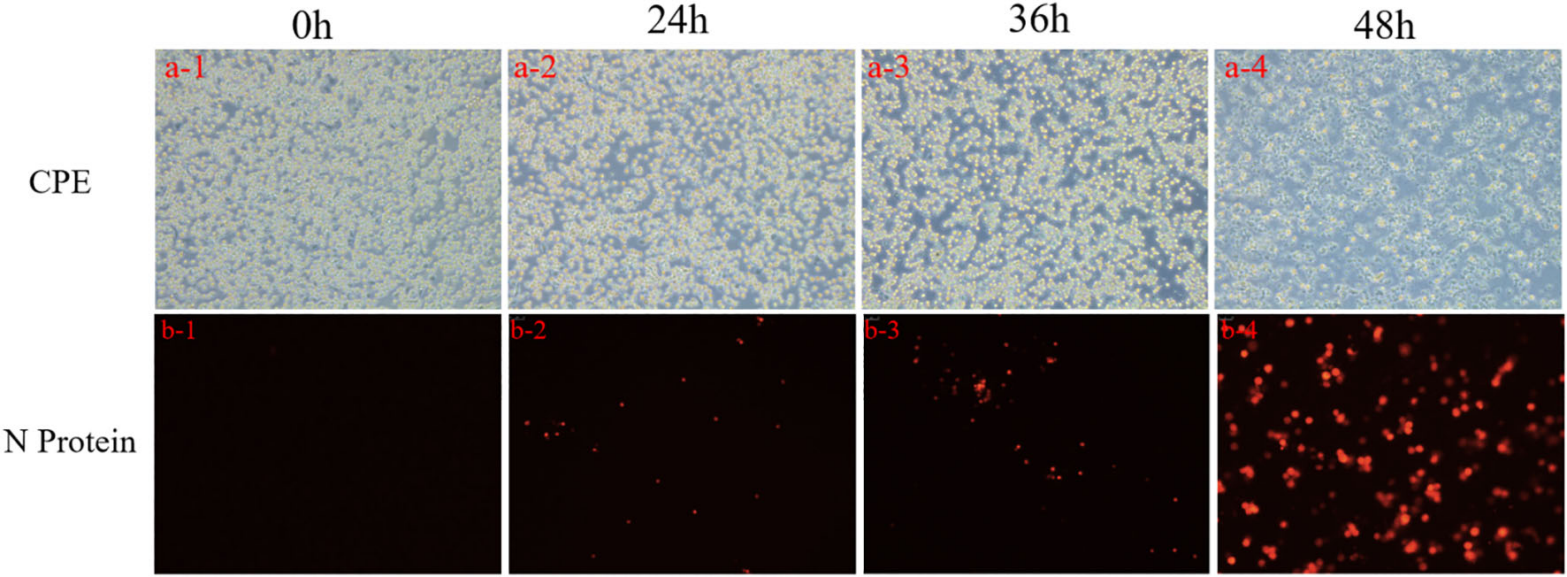
CPE and IFA identification of isolated strains on PAM cells. Note: a-1~a-4: Cell lesions observed at 0 h, 24 h, 36 h, and 48 h after inoculation of isolated strains onto PAM cells; b-1~b-4: Fluorescent labeling of N protein in cells at the four time points post-inoculation.

**Figure 3:**
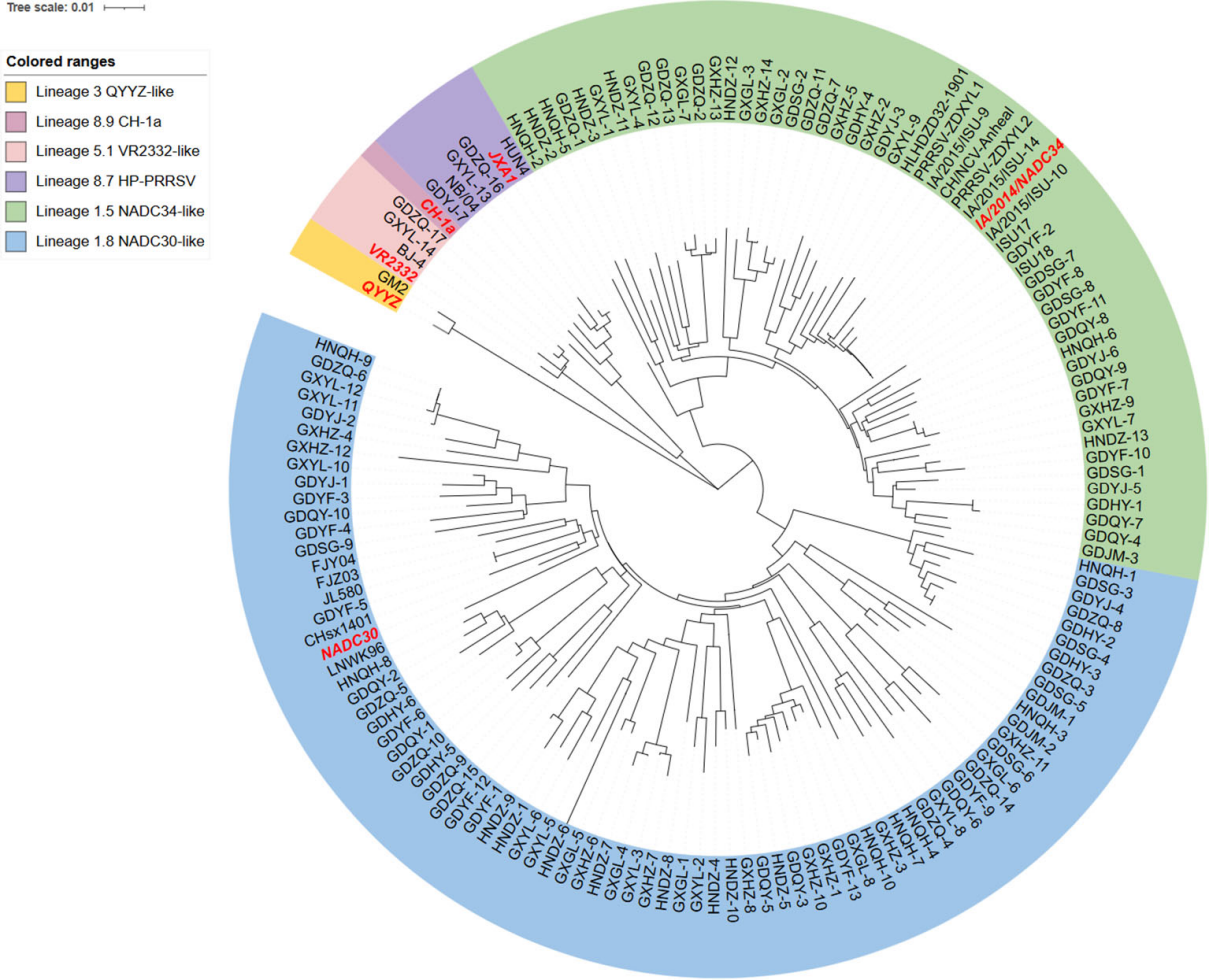
Phylogenetic analysis of GP5 sequences.

PRRSV-positive tissue samples were homogenized, filtered through a 0.22μm membrane, and inoculated onto PAMs under optimal growth conditions. CPE was observed post-inoculation. Significant CPE was observed after 36h in PAMs inoculated with PRRSV-positive samples [**Figure 2** (A1-4)]. Typical morphological changes included cell rounding, loss of monolayer adherence, cluster formation, and evident cell shrinkage. The degree of cell detachment progressively increased over time. Immunofluorescence assay (IFA) confirmed the presence of PRRSV-specific red fluorescence signals at 24, 36, and 48h post-inoculation. The fifth passage of PAMs showing typical CPE was freeze–thawed and subsequently inoculated into Marc-145 cells for virus amplification. CPE was first observed at 2 days post-inoculation, becoming more pronounced over time (**Figure 3**). Consequently, a PRRSV strain was successfully isolated and designated as PRRSV-DJW.

**Figure 4:**
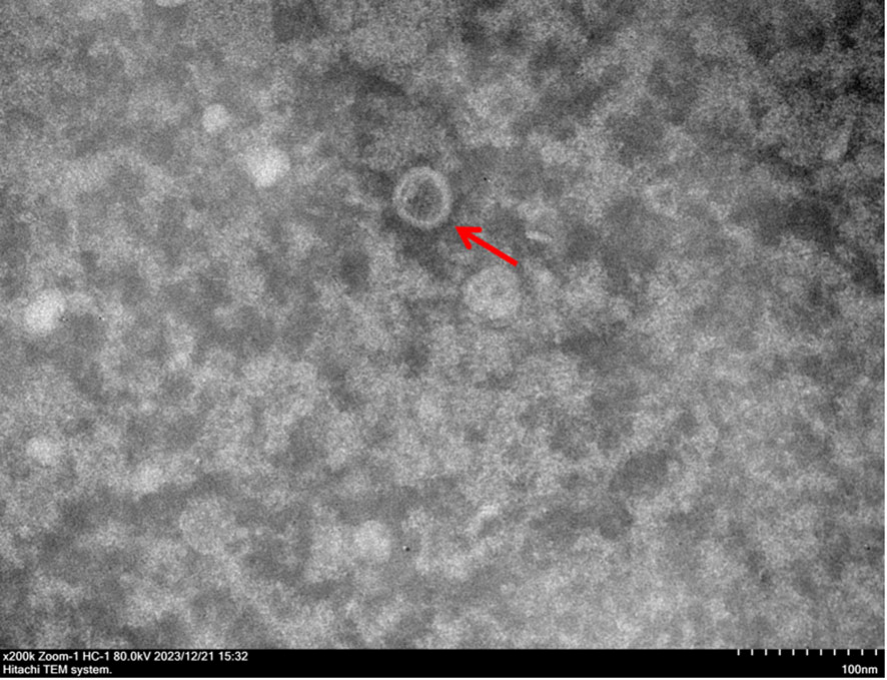
Morphological observation of PRRSV virus particles under electron microscopy.

Virus particles were enriched via ultracentrifugation and visualized by TEM. Under negative staining, virus particles exhibited a typical diameter of approximately 50–60 nm, intact morphology, and clear surface structures (**Figure 4**). These results confirmed successful viral replication and the stable expression of target genes in host cells.

#### 3.2.1 Whole-genome sequencing and genetic evolution analysis of the DJW strain

**Figure 5:**
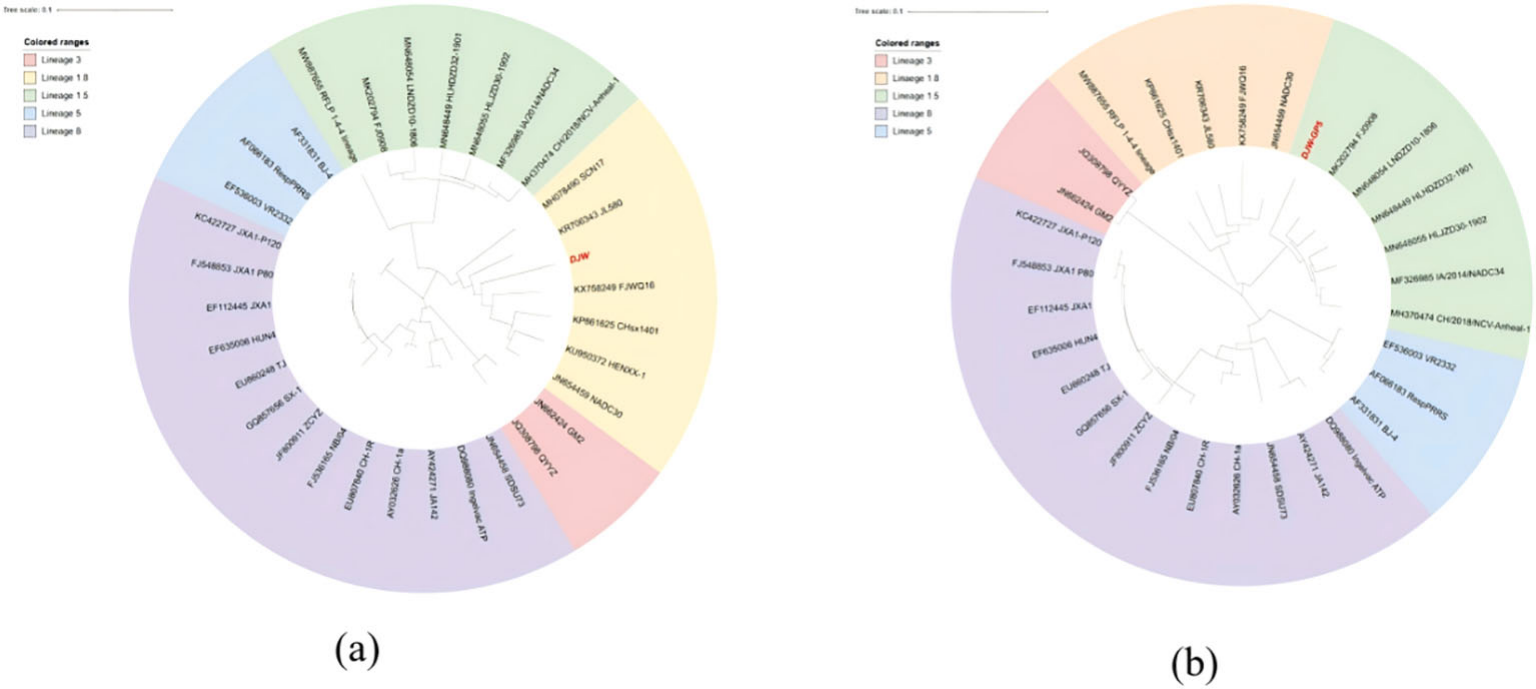
Phylogenetic analysis of DJW PRRSV: **(A)** Genetic evolution analysis based on full gene sequences; **(B)** Genetic evolution analysis based on ORF5 sequences.

The genomic DNA of the DJW strain was amplified using segment‑specific primers. The full-length genome sequence of the DJW strain was constructed using Seqman on DNASTAR. Phylogenetic trees based on *GP5* and the full genome were constructed using MEGA. Evolutionary analysis revealed that the DJW strain clustered within Lineage 1.8, if based on the full genome, but within Lineage 1.5, if based on *GP5*, indicating a possible recombination event (**Figure 5**).

#### 3.2.2 Recombination analysis of the DJW strain

**Figure 6:**
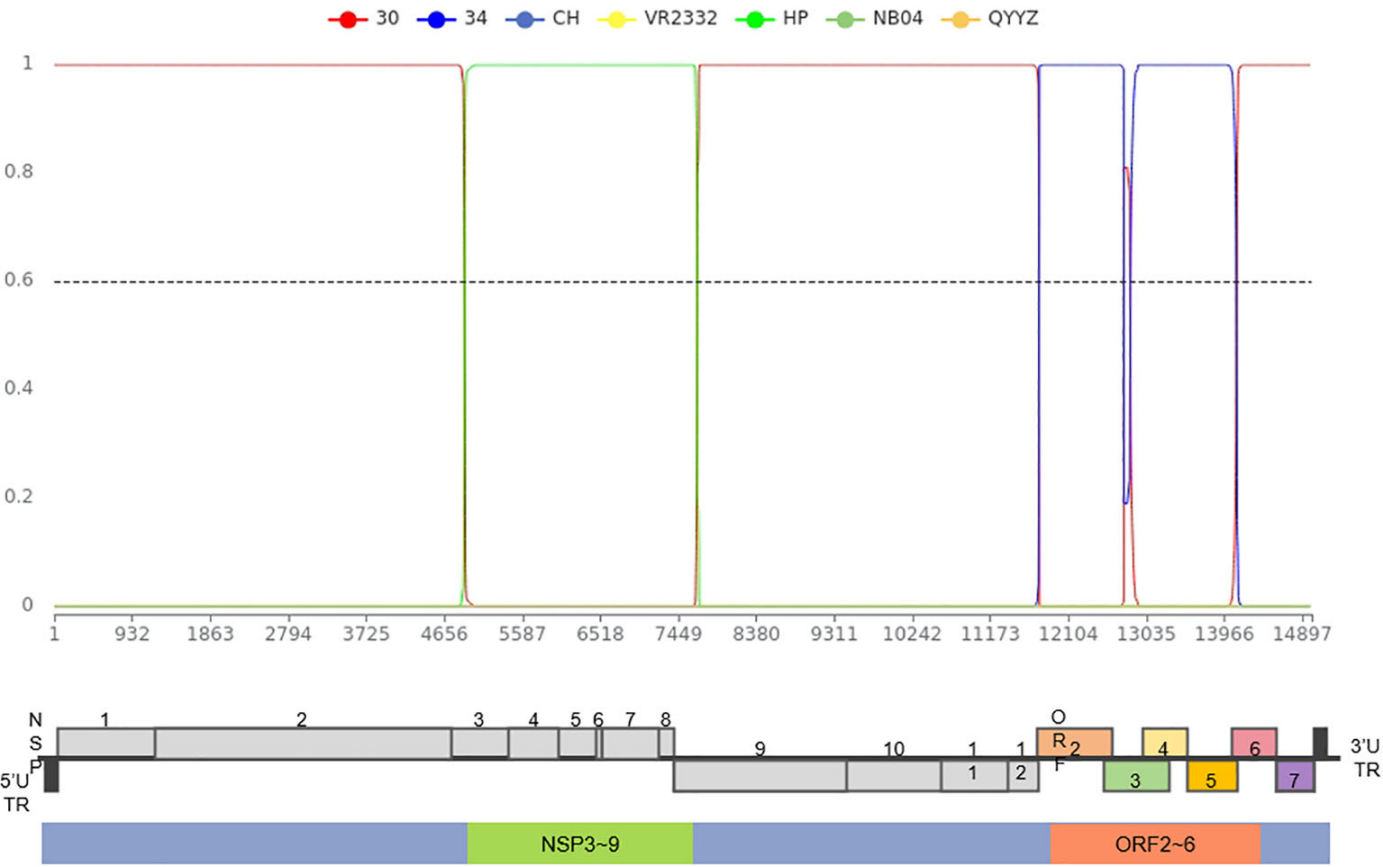
Recombination analysis of DJW strain.

Recombination analysis of the full-genome sequence of the DJW strain was performed using RDP4 software and validated using SimPlot. The DJW strain featured an NADC30 backbone, with recombination events occurring between nucleotides 4905–7675 (NSP3–NSP9) with an HP-PRRSV strain and between nucleotides 11751–14117 (ORF2–ORF6) with an NADC34 strain (**Figure 6**).

#### 3.2.3 GP5 sequence analysis

**Figure 7:**
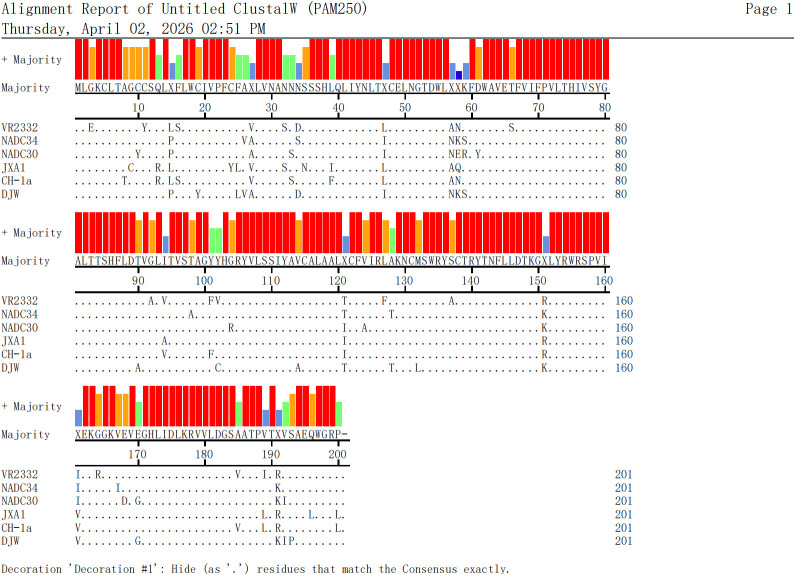
Alignment of GP5 amino acid sequences of DJW and representative PRRSV strains.

To evaluate the potential immune escape of the DJW strain, we aligned its GP5 amino acid sequence with those of representative PRRSV strains, including vaccine-related strains (VR2332, JXA1, CH-1a) and prevalent field strains (NADC30, NADC34). As shown in **Figure 7**, the primary neutralizing epitope (PNE, aa37–45) of DJW was identical to that of VR2332. However, a S32N substitution was observed in the decoy epitope region (aa28–36), and a V117A mutation was identified in the C-terminal neutralizing epitope region (aa115–125).

### 3.3 Pathogenicity analysis of the DJW strain in piglets

#### 3.3.1 Body temperature and weight gain monitoring

**Figure 8:**
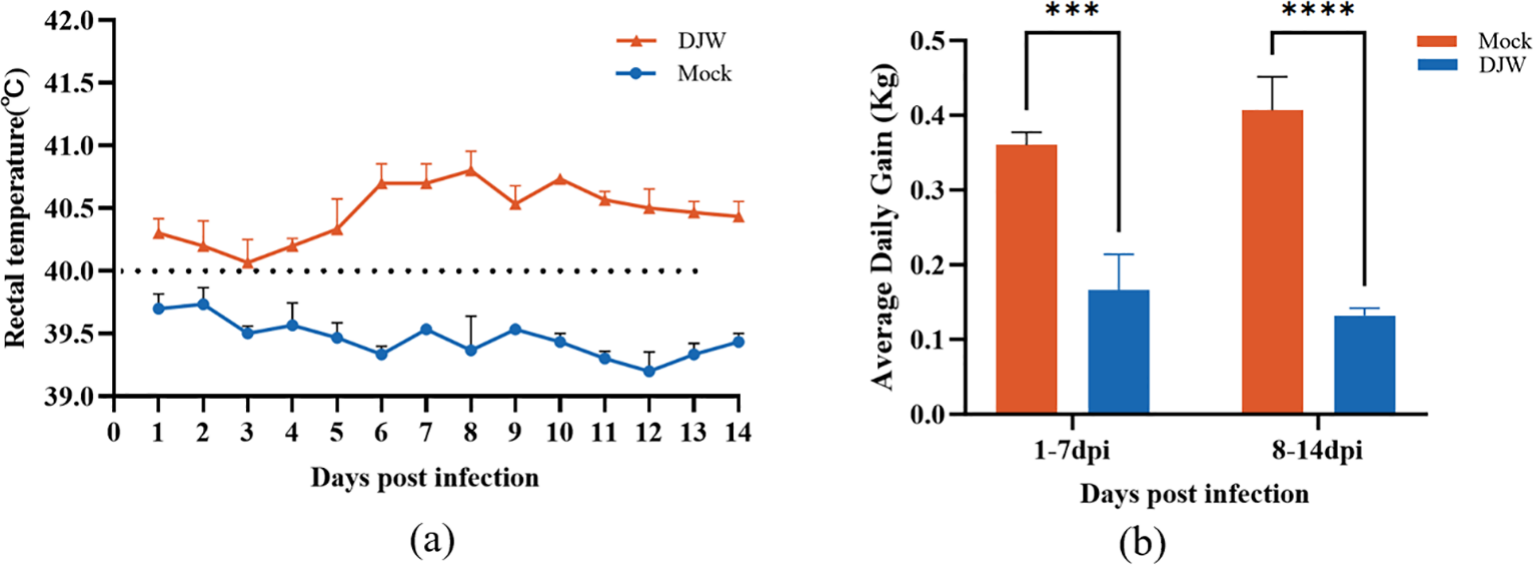
Monitoring of piglet body temperature and average daily weight gain. **(A)** Body temperature monitoring of piglets; **(B)** Average daily weight gain of piglets.

Body temperature monitoring indicated that DJW-inoculated piglets developed fever (≥40 °C) at 1 day post-inoculation (dpi). Body temperature peaked on 8 dpi and gradually decreased thereafter. In contrast, control piglets maintained normal body temperatures throughout the observation period. Weight measurements on 7 and 14 dpi revealed a lower average daily weight gain in the inoculated group than in the control group during both 1–7 dpi and 8–14 dpi (**Figure 8**).

#### 3.3.2 Viremia and viral shedding monitoring

**Figure 9:**
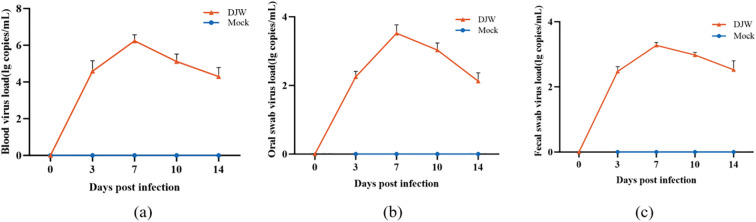
Piglet detoxification monitoring. **(A)** Viral RNA load in blood (viremia), **(B)** viral RNA shedding in oral swabs, and **(C)** viral RNA shedding in fecal swabs from piglets during the experimental period.

Blood, oropharyngeal, and anal swab samples were collected on 0, 3, 7, 10, and 14 dpi. In infected animals, viremia was first detectable at 3 dpi, reached a peak at 7 dpi, and gradually declined thereafter (**Figure 9A**). A similar temporal pattern was observed for viral shedding in both oropharyngeal and anal swabs, with viral loads also peaking at 7 dpi and subsequently decreasing through 14 dpi (**Figures 9B, C**). Notably, the peak viral load at 7 dpi was consistent across all three sample types, indicating this time point as the peak phase of viral replication and excretion. In contrast, no viral RNA was detected in any of the samples collected from the DJW or Mock control groups throughout the entire observation period.

#### 3.3.3 Antibody testing and survival rate

**Figure 10:**
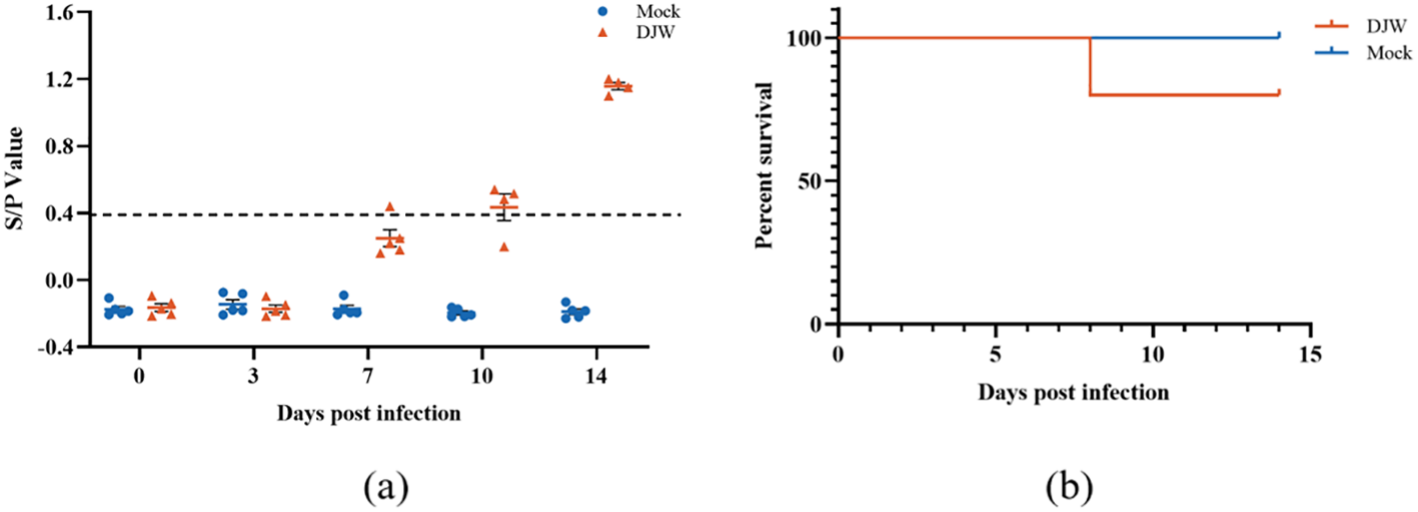
Antibody testing and survival rate. **(A)** Antibody titers in piglets; **(B)** Survival rate of piglets during the experimental period.

Serum anti-PRRSV-N antibody levels were monitored. In the DJW group, 75% of the surviving piglets seroconverted by 10 dpi, with all surviving piglets seroconverting by 14 dpi. In contrast, all piglets in the control group remained seronegative throughout the experiment (**Figure 10A**). One DJW-inoculated piglet died on 8 dpi, whereas all piglets in the control group survived until the end of the experiment (**Figure 10B**).

#### 3.3.4 Viral load in different tissues

**Figure 11:**
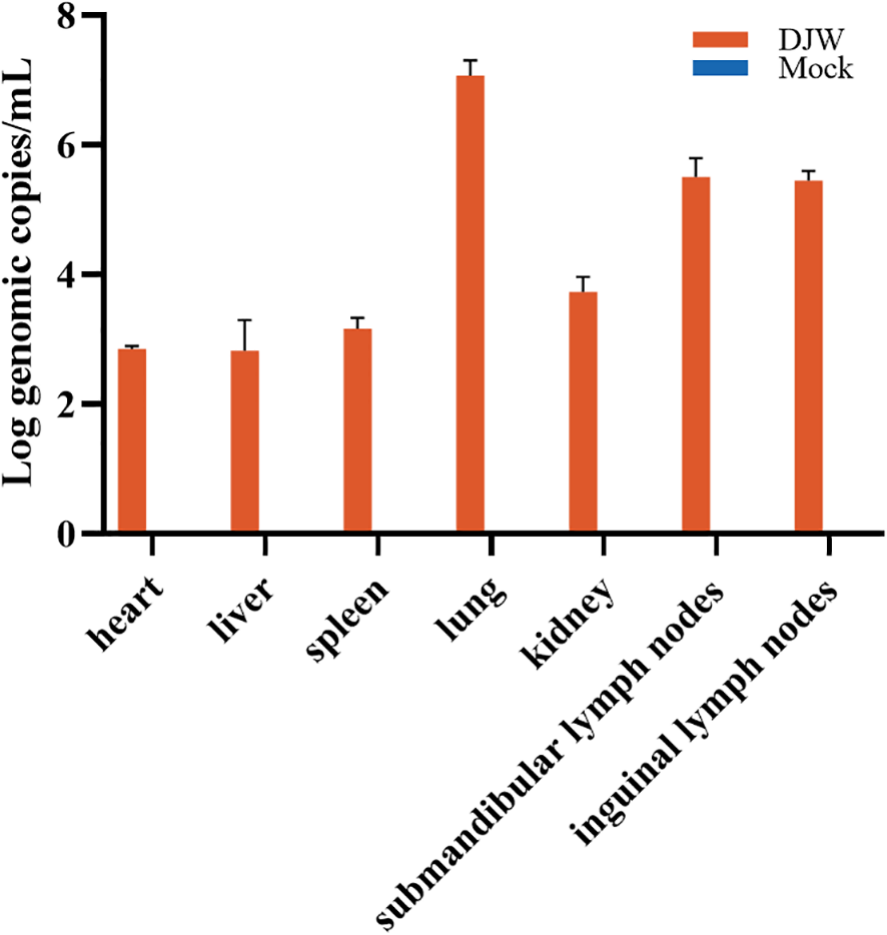
Detection of viral load in different tissues and organs.

At 14 dpi, the surviving piglets were euthanized, and various tissues were collected for viral load detection. The highest viral load was observed in the lungs (≥10^7^ copies/mL), followed by the submandibular and inguinal lymph nodes (10^5^–10^6^ copies/mL). No PRRSV units were detected in any tissues from the control group (**Figure 11**).

#### 3.3.5 Clinical autopsy and lung histopathological observation

**Figure 12:**
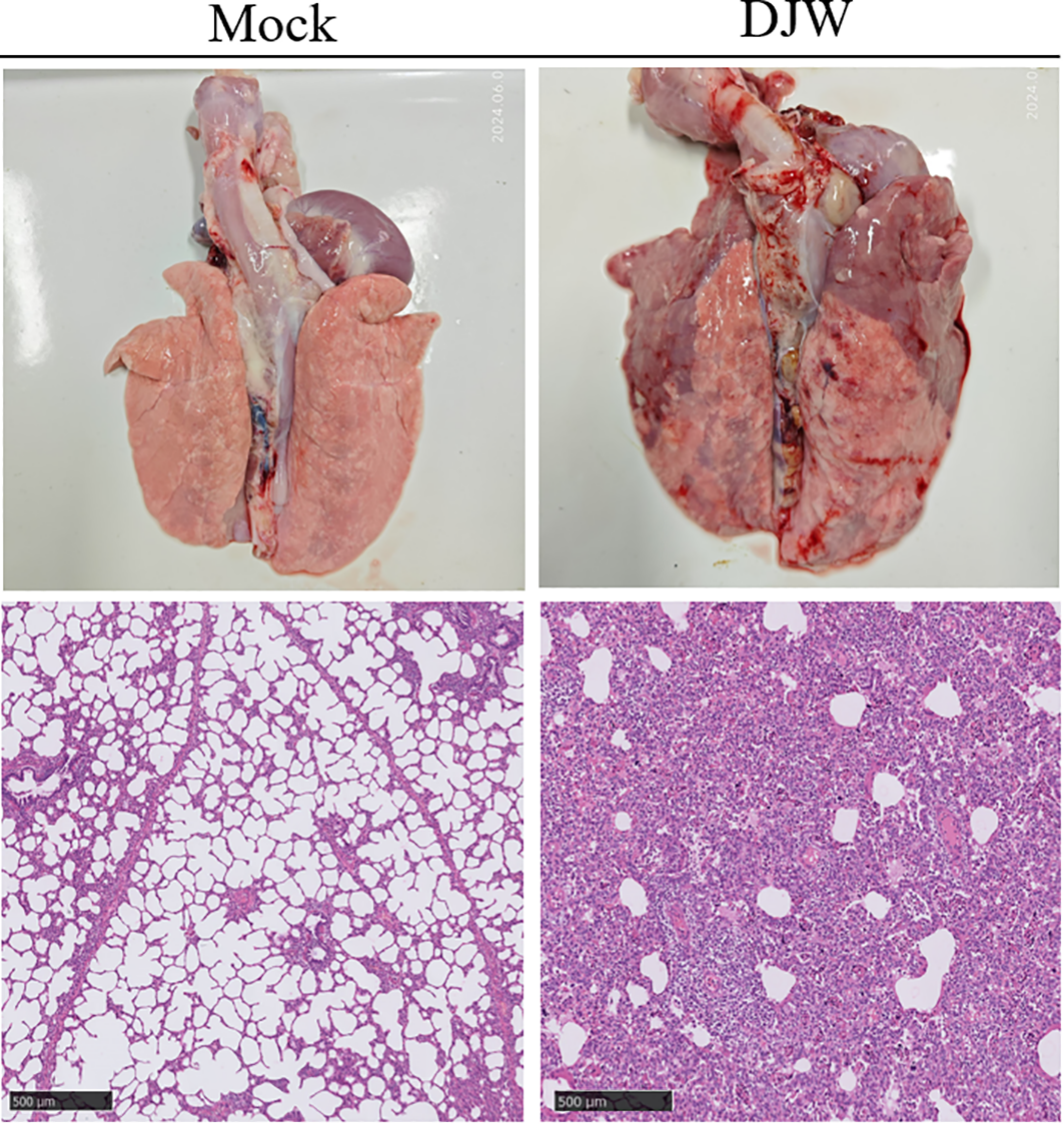
Clinical postmortem examination and histopathological observation of the lung in piglets.

Necropsy examination revealed severe macroscopic lesions in the lungs of the DJW group. Consolidation was noted in the cardiac, apical, and diaphragmatic lobes, presenting as firm tissue with dark-red discoloration. No macroscopic lesions were observed in the control group. Hematoxylin and eosin staining showed severe interstitial pneumonia in the DJW group, characterized by loss of normal alveolar airspace, alveolar septum thickening, dense alveolar interstitial tissue, and inflammatory exudation. In contrast, the control group exhibited normal lung morphology (**Figure 12**).

## 4 Discussion

PRRSV has been circulating in China for over two decades since its initial emergence and spread in the mid-1990s (Gao et al., 2017). The epidemiological progression of PRRSV in China has unfolded in three phases: the classical strain phase (1996–2006) (Tian et al., 2007), the HP-PRRSV phase (2006–2013) (Zhou et al., 2008), and the co-circulation phase of NADC30-like and HP-PRRSV strains from 2013 onward (Liu et al., 2021). NADC34-like strains have been increasingly detected across China in recent years. First identified in 2017, NADC34-like strains became highly prevalent between 2020 and 2022 (Oleksiewicz et al., 2001; Zhao et al., 2015; Wang et al., 2017). Moreover, NADC30-like viruses demonstrate a high propensity to recombine with HP-PRRSV, amplifying the genetic complexity of circulating strains (Zhang et al., 2016). This high variability and recombination capacity pose substantial challenges to PRRS management (Mateu and Diaz, 2008). In summary, the long-standing circulation (Tian et al., 2007), high mutation rate, and inadequate vaccine efficacy make PRRSV a persistent threat to the Chinese swine industry.

In this study, we collected 1, 139 clinical samples across 12 cities in three provinces in southern China. Of these, 463 tested positive for PRRSV, yielding a positivity rate of 40.6%. Genotypic characterization revealed that NADC30-like strains predominated (231/463; 49.9%), followed by NADC34-like (144/463, 31.1%), VR2332-like (46/463; 9.9%), HP-PRRSV-like (29/463; 6.3%), and QYYZ-like (13/463; 2.8%) strains. Phylogenetic analysis of 124 *ORF5* sequences revealed that 74 sequences (59.68%) clustered within Lineage 1.8, 45 (36.29%) within Lineage 1.5, while 2 (1.61%) and 3 (2.42%) within Lineages 5.1 and 8.7, respectively. Strains belonging to Lineages 3 and 8.9 were not detected. Geographically, positivity rates in Guangxi were 57.14% in 2016, 12.62% in 2017–2018, and 29.54% in 2020–2021. In Guangdong, reported rates were 16.60% (2007–2011), 32.54% (2010–2013), and 40% (2017–2018). In this study, the positivity rates were 41.5% (241/581) in Guangdong, 40.1% (131/326) in Guangxi, and 39.2% (91/232) in Hainan, indicating a stabilizing upward trend across these provinces. The currently circulating strains in southern China primarily belong to Lineage 1, which are mostly of the NADC30 and NADC34 sub-lineages. HP, QYYZ, and VR2332 strains are also in co-circulation. Although NADC30-like viruses remain dominant, the increasing detection of NADC34-like strains indicates that the latter has become a mainstream genotype in southern China.

The novel PRRSV-DJW strain was successfully isolated in this study. Whole-genome phylogenetic analysis classified the DJW strain within Lineage 1.8, while its *GP5* sequence clustered separately within Lineage 1.5. Recombination analysis further revealed that the strain exhibits a NADC30-derived backbone, with recombination events occurring in the NSP3–NSP9 regions (nucleotides 4905–7675) with an HP-PRRSV strain and in the ORF2–ORF6 regions (11751–14117 bp) with an NADC34-like strain, resulting in a triple-recombinant virus. This finding underscores the significant role of genomic recombination in PRRSV evolution (Zhang Z. et al., 2018).

Between 2015 and 2016, recombination events between NADC30 and Chinese HP-PRRSV strains produced several novel NADC30-like recombinants, which exhibited more complex virulence profiles than the original NADC30 strains (Liu et al., 2022; Guo et al., 2024). Current commercial vaccines have limited cross-protection efficacy, particularly against NADC30-related strains. For instance, the recombinant strain FJ1402, derived from NADC30 and HP-PRRSV, is highly pathogenic in piglets. Commercial vaccines, such as TJM-F92 and R98, only confer partial protection against FJ1402 (Zhang et al., 2016), highlighting the lack of full immunity. Therefore, characterizing the pathogenicity of recombinant PRRSV strains is essential for understanding their biological characteristics, despite the potential risk of live attenuated vaccines generating new recombinant viruses (Zhang Z. et al., 2018).

We analyzed its GP5 protein sequence in comparison with representative vaccine strains (VR2332, JXA1, CH-1a). The overall GP5 amino acid identity between DJW and these vaccine strains ranged from 85.6% to 88.1%, which is below the threshold typically associated with cross-protection. Notably, while the primary neutralizing epitope (PNE, aa37–45) of DJW was identical to that of VR2332 (SHLQLIYNL), we identified a S32N substitution in the decoy epitope region (aa28–36) and a unique V117A mutation in the C-terminal neutralizing epitope region (aa115–125). The decoy epitope is known to induce non-neutralizing antibodies that may divert immune responses, and the V117A substitution may alter conformational epitope integrity. These findings suggest that the protective efficacy of current vaccines may be compromised, a conclusion that requires confirmation through *in vivo* studies.

The presence of recombination events indicates the co-circulation of multiple PRRSV strains within swine populations, creating a potential perpetuating cycle of viral recombination, which is a key driver of viral evolution, virulence modulation, and immune escape (Ansari et al., 2006). Evidently, the generation of recombinant strains further complicate PRRS control (Renukaradhya et al., 2015). The virulence of the DJW strain was evaluated in a pathogenicity study conducted in piglets. Infection resulted in fever, viral shedding, growth retardation, lung pathology, and one death observed in the challenge group (1/5), clinical manifestations indicative of moderate to high pathogenicity. Previous studies have found considerable variability in the pathogenicity of recombinant strains (Cha et al., 2004; Song et al., 2020; Yuan et al., 2022; Xu et al., 2024). For example, the GS2022 strain, a recombinant of NADC30-like and JXA1-like viruses, caused mild clinical signs, including transient fever, respiratory distress, and gross and histopathological lesions in the lungs and lymph nodes (Guo et al., 2024). The HLJDZD32–1901 strain induced mild respiratory signs and slightly increased body temperature (Song et al., 2020), whereas the JS2021 strain exhibited high pathogenicity, causing persistent fever and mortality (Yuan et al., 2022). Interestingly, the SDVD-NMG2023 strain, which otherwise shares a similar recombination pattern with DJW (triple recombination among JXA1, NADC30, and NADC34, with recombination in the NSP3–NSP8 regions with JXA1), demonstrated low pathogenicity, causing low-grade fever, mild lung lesions, and minimal impact on growth (Brown et al., 2009; Yang et al., 2024),. In contrast, PRRSV/CN/FJGD01/2021, another triple-recombinant mosaic, generated high viral loads, persistent fever, significant weight loss, moderate respiratory signs, and severe pulmonary histopathology (Liu et al., 2022). These findings demonstrate the profound genetic variability and diverse recombination patterns across PRRSV strains, which result in substantial divergence in pathogenicity (Yuan et al., 2022; Choi et al., 2023). Developing effective management strategies tailored to the current complex epidemiological landscape and varying virulence profiles of PRRSV remains an urgent and formidable challenge (Zhou et al., 2021).

A limitation of this study is the small sample size (n=5 per group) used in the piglet challenge experiment. Although the observed clinical signs, viral shedding, and mortality (one death in the challenge group, 1/5) indicate high virulence, the small cohort precludes robust statistical generalization of mortality rates. Future studies with larger animal numbers are warranted to further characterize the pathogenicity of the DJW strain.

## 5 Conclusion

This study confirms the ongoing dominance and rapid evolution of PRRSV Lineage 1 strains in southern China, primarily driven by NADC30-like and NADC34-like variants. The isolation of the novel triple-recombinant mosaic strain DJW, and its demonstration of severe clinical signs and pathology, in piglets including severe respiratory disease and one death observed in the challenge group (1/5), underscores the critical role of recombination in generating new, moderately to highly virulent PRRSV variants. This finding validates the urgent need for vigilant surveillance and updated control strategies.

## Data Availability

The datasets presented in this study can be found in online repositories. The names of the repository/repositories and accession number(s) can be found in the article/supplementary material.
